# A Label Free Colorimetric Assay for the Detection of Active Botulinum Neurotoxin Type A by SNAP-25 Conjugated Colloidal Gold

**DOI:** 10.3390/toxins5081381

**Published:** 2013-08-06

**Authors:** Jennifer Halliwell, Christopher Gwenin

**Affiliations:** School of Chemistry, Bangor University, Bangor, Gwynedd, Wales LL57 2DG, UK; E-Mail: chu60f@bangor.ac.uk

**Keywords:** botulinum neurotoxin, SNAP-25, localised surface plasmon resonance, colloidal gold

## Abstract

Botulinum neurotoxins are one of the most potent toxins known to man. Current methods of detection involve the quantification of the toxin but do not take into account the percentage of the toxin that is active. At present the assay used for monitoring the activity of the toxin is the mouse bioassay, which is lengthy and has ethical issues due to the use of live animals. This report demonstrates a novel assay that utilises the endopeptidase activity of the toxin to detect Botulinum neurotoxin in a pharmaceutical sample. The cleaving of SNAP-25 is monitored *via* UV-Visible spectroscopy with a limit of detection of 373 fg/mL and has been further developed into a high throughput method using a microplate reader detecting down to 600 fg/mL of active toxin. The results show clear differences between the toxin product and the placebo, which contains the pharmaceutical excipients human serum albumin and lactose, showing that the assay detects the active form of the toxin.

## 1. Introduction

The method currently used for determining the concentration of toxin in a sample is the mouse bioassay. This assay involves injecting the mice with a sample of the toxin and observing them for symptoms of the disease over a period of up to four days [1]. The mouse bioassay is able to detect 10 pg/mL of the toxin; however, it is very lengthy, expensive and has ethical problems in the use of live animals [2]. 

There is demand for alternative methods to reduce and replace the use of live animals and many botulinum assays are currently being developed including ELISA and fluorescent endopeptidase assays which outperform the mouse bioassay and takes 1–3 h [3,4,5]. An assay which outperforms current limits of detection and takes minutes to perform would fit a gap in the market having uses in the pharmaceutical industry for batch quality control, as a point of care clinical sensor in suspect botulism cases as well as in biosecurity applications [6].

Botulinum neurotoxins are zinc dependent endopeptidases which cleave the soluble *N*-ethylmaleimide-sensitive attachment protein receptor (SNARE) proteins [7]. The toxin has a mass of 150 kDa and is split into two domains; the heavy chain (100 kDa) which is responsible for uptake into cells and the catalytic light chain (50 kDa) linked to the heavy chain *via* a disulfide bond [8]. Due to the paralysing effect of the toxin it is often used in low doses to treat conditions such as cervical dystonia and blepharospasm [9].

This assay utilises colloidal gold to produce a visible colour change on addition of sodium chloride. Colloidal gold has been used to develop a wide range of sensors [10,11,12]. Specific wavelengths of light excite the conduction electrons on the metal surface causing them to oscillate. This oscillation is known as localised surface plasmon resonance (LSPR) and is responsible for the absorption of certain wavelengths of light producing characteristic UV-visible spectrums with a typical absorption peak occurring at around 520 nm for colloids with a diameter of 10–50 nm [13]. The nanoparticles in colloidal gold are negatively charged and repel each other and hence remain colloidal in nature and appear red in colour, when salt is added these negative charges are masked and the particles aggregate together [14]. This aggregation causes a decrease in interparticle distance allowing the plasma modes to couple together causing the particles to absorb at a longer wavelength, typically around 700 nm turning the colloidal solution blue [15]. A protein layer covalently bonded onto the gold surface protects the nanoparticles from the ionic effects of the salt, maintaining the red colour and keeping them in solution [16]. 

Botulinum neurotoxin A selectively cleaves the SNARE protein SNAP-25 between residues 197–198 [17]. SNAP-25 has four cysteine amino acids located at positions 85, 88, 90 and 92; these residues are used to attach to the neural plasma membrane by undergoing palmitoylation, causing the middle cysteine section to be anchored down and the helix chains pointing away from the surface [18,19]. Cysteine is able to form a covalent sulfur-gold bond, therefore, SNAP-25’s native cysteine residues can be used to attach the protein to gold nanoparticles [20]. Using SNAP-25’s native cysteine allows the protein to be orientated correctly, as it would be in the body, so the toxin is not inhibited from reaching the multiple recognition sites [21]. On the addition of the toxin nine amino acids will be cleaved off the C-terminus of the protein, decreasing the protective layer around the nanoparticles making them more susceptible to the salt turning the solution blue.

## 2. Results and Discussion

### 2.1. Coating of Colloidal Gold

**Figure 1:**
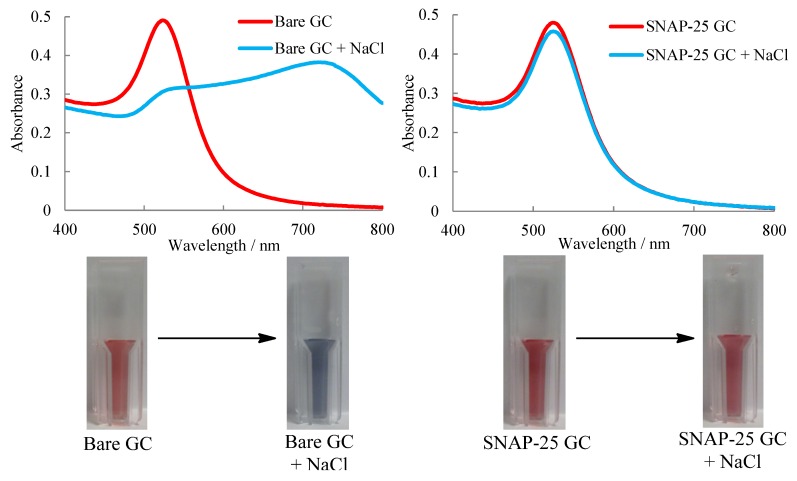
UV-Visible Spectroscopy showing uncoated (**left**) and coated (**right**) gold colloids (GC) and the addition of salt causing aggregation of the uncoated particles and cuvette images showing the visible colour change.

UV-Visible spectrum of coated and uncoated gold particles were measured before and after salt was added to prove that the SNAP-25 bound with the gold surface (Figure 1). 

Both uncoated and coated nanoparticles showed plasmon absorbance at around 525 nm as expected for gold colloids of 20 nm diameter. On addition of salt the uncoated gold nanoparticles aggregate and turn blue in colour due to the coupling of the localised plasmon resonance increasing the wavelength to around 700 nm. The coated gold nanoparticles show no red shift and only a slight decrease in absorbance at 525 nm which can be attributed to the dilution of the sample with the salt solution. Thus proving that the particles were sufficiently coated with SNAP-25 as the electrostatic repulsion of the particles was preserved.

**Figure 2:**
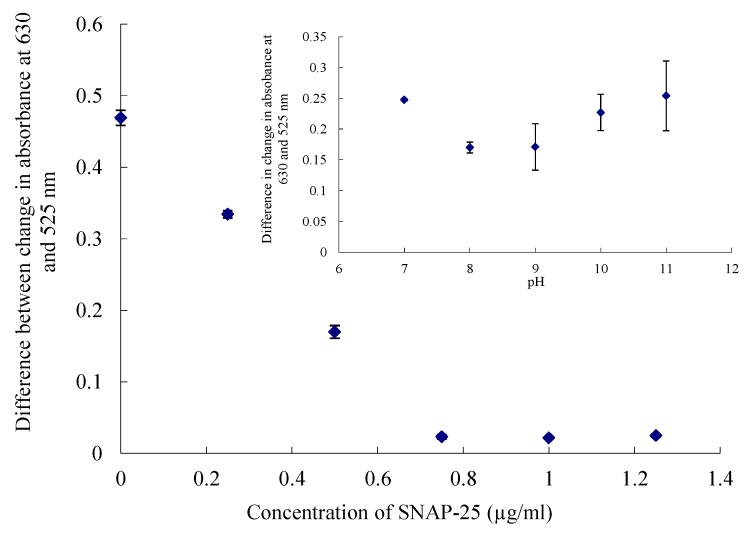
Difference in change in absorbance at 630 and 525 nm on addition of salt with concentration of SNAP-25. (Insert) surface covered with 0.5 µg/mL of SNAP-25 at different pH values. Errors calculated to ± 1SD.

The coating of the colloids was repeated with a range of SNAP-25 concentrations and different pH buffers in the absence of toxin to find the optimum conditions for coating (Figure 2).

Where the absorbance at the two wavelengths showed little change the particles were considered to be stable [22]. The particles were coated with 0.5 µg/mL SNAP-25 for the UV-visible and microplate assay, as, at this concentration, the particles are stable enough so that when the toxin cleaves the nine amino acids of the SNAP-25 they become susceptible to the effects of the salt. If the concentration of the SNAP-25 was too high the particles were too stable and no change in absorbance could be seen. The particles became unstable at low pH values, as shown in the insert in Figure 2, and it has been reported that an alkaline pH leads to the separation of the toxin from its associated proteins leading to a decrease in endopeptidase activity; hence a pH of 8 was chosen for this data set [23]. 

### 2.2. SDS-PAGE

**Figure 3:**
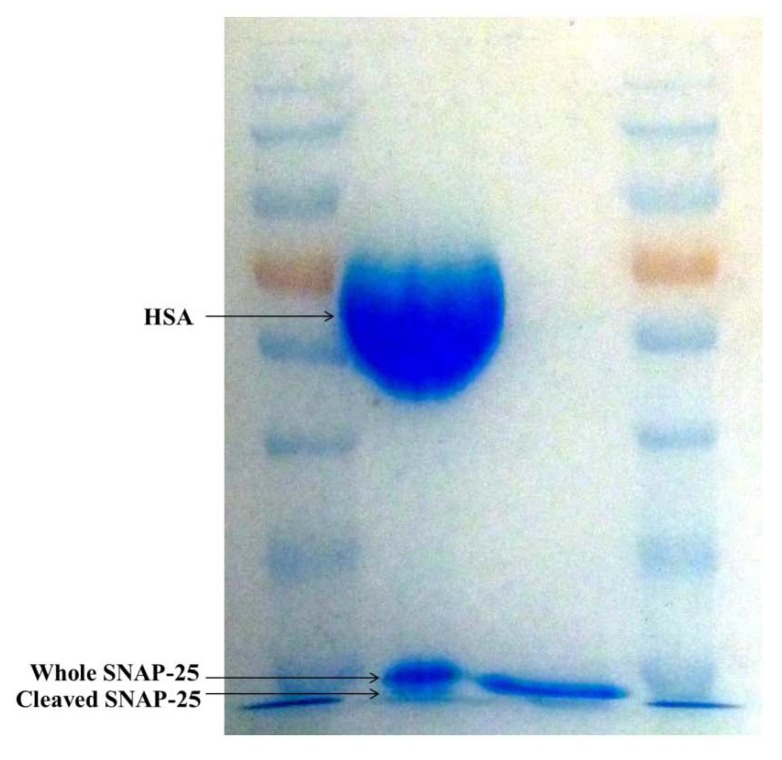
SDS-PAGE gel showing cleaved and whole SNAP-25.

The toxin and SNAP-25 mixture was loaded into the left hand lane with SNAP-25 alone in the right. As there is only 4.35 ng of toxin in Dysport^®^ it does not appear on the gel; however a large band appears at 67 kDa which is the HSA in the toxin product. The lane on the left shows two bands around 25 kDa which correspond to the whole and cleaved SNAP-25; as it is only reduced by 1 kDa it can be difficult to see the two separate bands. However when compared to the SNAP-25 which has not been incubated in the right hand lane the two bands are obvious. SDS-PAGE was used to confirm that the SNAP-25 substrate used in the assay is cleaved by the toxin (Figure 3).

### 2.3. UV-Visible Spectroscopy Assay

**Figure 4:**
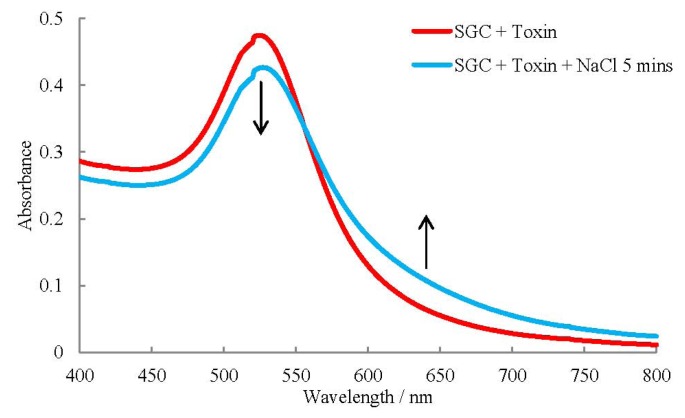
UV-Visible Spectrum of SNAP-25 coated gold colloids (SGC) incubated with 1 pg/mL botulinum neurotoxin before NaCl was added and incubated for five minutes.

Once the optimum conditions for the coating of the particles and the pH of the buffer was determined the assay was performed using UV-visible spectroscopy to measure the change in absorbance. A typical spectrum is shown in Figure 4.

The spectra for the coated particles with toxin before and after the addition of NaCl show no change in absorbance. After incubating with NaCl for five minutes there is a decrease in absorbance predominantly at 500–550 nm and an increase at around 575–800 nm with the greatest changes occurring at 525 and 630 nm. These results are remarkable considering only 9 amino acids are cleaved off SNAP-25 and 1 µL of sample used. Low sample volumes are used due to the regeneration of the toxins active site after a SNAP-25 cut allowing it to cleave multiple SNAP-25 molecules [24].

**Figure 5:**
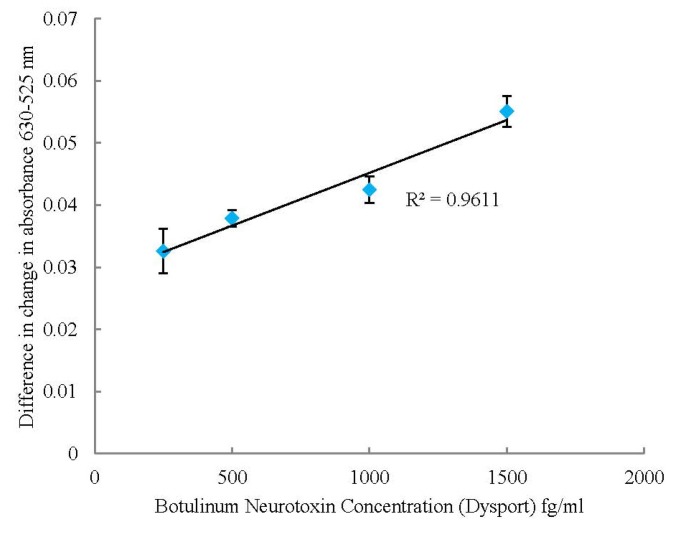
Correlation graph showing difference in change in absorbance at 630 and 525 nm for a range of Botulinum Neurotoxin concentrations using the UV-Visible Spectrometer. Errors calculated to ± 1SD.

A range of toxin concentrations and the placebo samples were measured in triplicate with the volume of sample added remaining consistent. The change in absorbance’s at 630 nm and at 525 nm were calculated then the differences between these two figures used to produce a correlation graph; Figure 5.

The graph shows good correlation for the toxin samples over a range of 250–1500 fg/mL, with the results plateauing at the upper and lower limit, with a calculated limit of detection of 373 fg/mL [25]. The denatured sample, as prepared in Section 3.2, and the placebo, human serum albumin (125 µg) and lactose (2.5 mg), which are the excipients of the Dysport^®^ product supplied by IPSEN, showed a lower response at 0.0242 and 0.0236 respectively. This illustrates that the excipients do not cause false positive results and the assay is detecting active toxin within the range tested. This assay is more sensitive than the mouse bioassay and much quicker with each sample only taking 10 min for analysis.

### 2.4. Microplate Assay

**Figure 6:**
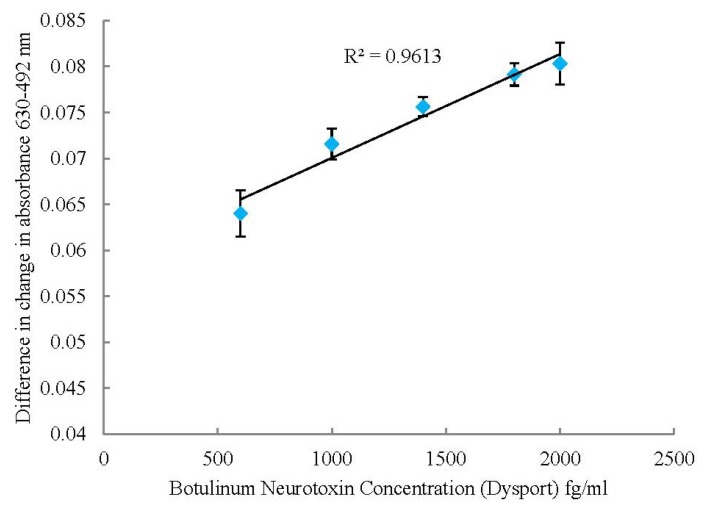
Correlation graph showing difference in change in absorbance at 630 and 492 nm for a range of Botulinum Neurotoxin concentrations using the microplate reader. Errors calculated to ± 1SD.

The assay was repeated using 96 well plates again with each sample being analysed in triplicate. The change in absorbance’s at 630 nm and 492 nm were calculated then the difference between these two figures used to produce the graph; Figure 6. 

The absorbance was measured at 492 nm rather than 525 nm as in the UV-visible spectroscopy assay due to the availability of wavelength filters for the microplate reader. This changed the detection range to 600–2000 fg/mL with a calculated limit of detection of 278 fg/mL, again with the results plateauing at the upper and lower limits. The linear detection range was slightly higher than the UV-visible spectrometry but still lower than the mouse bioassay. Using the microplate reader allows a much higher throughput; taking seven minutes for 96 samples. The response for the placebo and denatured toxin samples were again much lower than the toxin samples at 0.0523 and 0.0572 respectively showing this assay is detecting the active form of the toxin.

## 3. Methods and Materials

Colloidal gold (20 nm, 7 × 10^11^ particles/mL) was purchased from BBInternational, SNAP-25 was from Stratech Scientific, Monopotassium phosphate, Dipotassium phosphate, Zinc Chloride, Dithiothreitol, Tween-20 and HEPES were purchased from Sigma Aldrich and Sodium Chloride was bought from Fisher Scientific. Dysport^®^ samples containing lyophilised *Clostridium Botulinum* Type A toxin-haemaggluttinin complex (4.35 ng), human serum albumin (125 µg) and lactose (2.5 mg) and placebos containing the excipients from Dysport^®^ were supplied by IPSEN Biopharm. Water was purified and had a nominal resistivity of 18 MΩ cm at 25 °C.

### 3.1. Coating Colloidal Gold

The colloidal gold solution was mixed in a 1:1 ratio with phosphate buffer (10 mM, pH 8) and placed on an orbital shaker at 37 °C to equilibrate. SNAP-25 solution was incubated at 37 °C before being added (500 ng/mL) to the gold solution and left on the shaker overnight. All solutions were kept at 37 °C prior to use, all incubations were also performed at this temperature.

The concentration of SNAP-25 and the pH of the buffer were determined by coating colloids in a range of concentrations in buffers of varying pH. Phosphate buffer was used for pH 7–8 and a sodium carbonate/bicarbonate buffer was used for pH 9–11. Aliquots of these solutions (960 µL) were transferred to cuvettes and the absorbance from 400 to 800 nm measured. NaCl (4 M, 40 µL) was then added, incubated for 5 min and the absorbance re-measured.

### 3.2. Pre-treatment of Samples

Before analysis samples were diluted in a digestion buffer (20 µL) of ZnCl_2_ (0.5 mM), Dithiothreitol (2 mM), HEPES (50 mM) and Tween-20 (0.05%) at pH 7.4 and incubated for an hour at 37 °C [26]. This reduced any toxin present in the sample to the active form by separating the catalytic light chain from the heavy chain.

Denatured samples were produced by incubating Dysport^®^ samples at 95 °C for 15 min [27].

### 3.3. SDS-PAGE

Toxin was pre-treated before SNAP-25 was added to a final concentration of 3.3 µg/µL and incubated at 37 °C for an hour. The toxin/SNAP-25 and SNAP-25 sample were added to a 10% SDS-PAGE gel and run at 100 V.

### 3.4. UV-Visible Spectroscopy Assay

The SNAP-25 gold conjugate (960 µL) was added to a cuvette and the absorbance read from 400–800 nm using a Jasco V-550 UV-Visible Spectrometer. The sample (1 µL) was then added and the absorbance re-measured the solution was then allowed to incubate for 5 min. NaCl (4 M, 40 µL) was added and the absorbance measurement repeated. The solution was left to incubate for a further 5 min before a final reading was taken. All measurements were recorded against a reference sample of phosphate buffer with the background absorbance removed.

### 3.5. Microplate Assay

Samples (0.5 μL) were added to the wells of a microliter plate and SNAP-25 gold conjugate (374 µL) added to each well before the absorbance at 492 and 630 nm was measured using a Labtech LT-4000 microplate reader. The plate was then incubated for 2 min before NaCl was added (4 M, 15 µL) and incubated for 5 min. before the absorbance was re-measured. 

## 4. Conclusions

Many assays currently being developed, such as the ELISA assay, only detect the presence of the toxin and are unable to measure the proteolytic activity, so do not differentiate between active and denatured toxin [28]. By measuring the colour change after the toxin has cleaved the SNAP-25, our assay detects only active botulinum neurotoxin making it highly useful for the pharmaceutical industry. This is particularly relevant when the low detection limit of 250 fg/mL is considered, which is lower than the mouse bioassay and requires only 0.5–1 µL per sample, making it safer and cheaper to be run. These assays detect much lower amounts of toxin than the amount typically contained in pharmaceutical products, around 4 ng, meaning that a set of serial dilutions would need to be performed to analyse the products correctly. This is simple to perform and would only add a few minutes onto the overall analysis time. 

This assay may also be used as a point-of-care sensor and as a screening step in the food industry with the inclusion of sample purification steps such as immunochromatography and centrifugation [29,30].

With the transfer of the assay onto a microplate reader 96, samples can be processed in seven minutes allowing for high throughput analysis. This also leads to the possibility of automating the assay and using plates with more wells, further increasing output.

SNAP-25 is cleaved by serotypes A, C and E, as stated in the literature. By using SNAP-25 as the substrate this assay currently can only detect these serotypes [31]. This assay will be further developed to detect other serotypes by modifying the gold surface with VAMP and Syntaxin through the sulfur on the methionine side chain [32].

Many botulinum neurotoxin assays do not advance from the development stage due to interference from excipients commonly found in toxin drug products such as HSA [33]. This assay has been developed using Dysport^®^ and a placebo which contains HSA and lactose. The results show good correlation and the placebo has a much lower response proving this assay is not affected by the excipients [34].
